# Characteristics and Performances of a Nanostructured Material for Passive Samplers of Gaseous Hg

**DOI:** 10.3390/s20216021

**Published:** 2020-10-23

**Authors:** Joshua Avossa, Fabrizio De Cesare, Paolo Papa, Emiliano Zampetti, Andrea Bearzotti, Marcello Marelli, Nicola Pirrone, Antonella Macagnano

**Affiliations:** 1 Institute of Atmospheric Pollution Research—National Research Council, Research Area of Rome 1, Via Salaria km 23,600, Monterotondo, 00016 Rome, Italy; joshua.avossa@empa.ch (J.A.); decesare@unitus.it (F.D.C.); p.papa@iia.cnr.it (P.P.); e.zampetti@iia.cnr.it (E.Z.); a.bearzotti@iia.cnr.it (A.B.); 2 Laboratory for Biomimetic Membranes and Textiles, Empa, Swiss Federal Laboratories for Materials Science and Technology, Lerchenfeldstrasse 5, CH-9014 St. Gallen, Switzerland; 3 Department of Innovation in Biological Systems, Food and Forestry (DIBAF), Via S. Camillo de Lellis, University of Tuscia, 00100 Viterbo, Italy; 4 Institute of Chemical Sciences and Technologies “Giulio Natta” (SCITEC)—National Research Council, c/o Area di Ricerca di Milano 1, Sede Fantoli, Via Fantoli 16/15, 20138 Milano, Italy; marcello.marelli@scitec.cnr.it; 5 Institute of Atmospheric Pollution Research—National Research Council, Division of Rende, UNICAL Polifuzionale, 87036 Rende, Italy; pirrone@iia.cnr.it

**Keywords:** gaseous mercury pollution, Au-TiO_2_ nanoparticles, passive sampler, axial diffusion, thermal desorption, monitoring campaign

## Abstract

Passive air samplers (PASs) have been used for mapping gaseous mercury concentration in extensive areas. In this work, an easy-to-use and -prepare gold nanoparticle (NP)-based PAS has been investigated. The PAS is constituted of a microfibrous quartz disk filter impregnated of gold NP photo-growth on TiO_2_ NPs (Au@TiO_2_) and used as gaseous mercury adsorbing material. The disk was housed in a cylinder glass container and subjected to an axial diffusive sampling. The adsorbed mercury was measured by thermal desorption using a Tekran^®^ instrument. Different amounts of Au@TiO_2_ (ranging between 4.0 and 4.0 × 10^−3^ mg) were deposited by drop-casting onto the fibrous substrate and assessed for about 1 year of deployment in outdoor environment with a mercury concentration mean of about 1.24 ± 0.32 ng/m^3^ in order to optimize the adsorbing layer. PASs showed a linear relation of the adsorbed mercury as a function of time with a rate of 18.5 ± 0.4 pg/day (≈1.5% of the gaseous concentration per day). However, only the PAS with 4 mg of Au@TiO_2_, provided with a surface density of about 3.26 × 10^−2^ mg/mm^2^ and 50 μm thick inside the fibrous quartz, kept stability in working, with a constant sampling rate (SR) (0.0138 ± 0.0005 m^3^/day) over an outdoor monitoring experimental campaign of about 1 year. On the other hand, higher sampling rates have been found when PASs were deployed for a few days, making these tools also effective for one-day monitoring. Furthermore, these PASs were used and re-used after each thermal desorption to confirm the chance to reuse such structured layers within their samplers, thus supporting the purpose to design inexpensive, compact and portable air pollutant sampling devices, ideal for assessing both personal and environmental exposures. During the whole deployment, PASs were aided by simultaneous Tekran^®^ measurements.

## 1. Introduction

Mercury (Hg) is a persistent bio-accumulative toxic metal [1] existing in many different forms in the environment. It continuously cycles between the atmosphere, soil and ocean changing form and stability: when mercury is released into the atmosphere, it can remain there or be transferred to soil or oceans, and eventually return to the atmosphere. Gaseous Elemental Mercury (GEM) is the most stable form of Hg (abbreviated as Hg(0) or Hg^0^)**.** Hg^0^ is the most abundant form in ambient air, with a residence time spanning from months until about 1 year [2]. Hg^0^ can be oxidized in the troposphere [3] forming different divalent species, like HgCl_2_, HgBr_2_, HgO, HgBrOH named Gaseous Oxidized Mercury (GOM) [3,4,5,6]. GEM and GOM represent the total amount of gaseous mercury species, and for this reason, they are named as Total Gaseous Mercury (TGM). Mercury chemical species adsorbed on particulate matter form the so-called Particulate Bound Mercury (PBM) [7]. Compared to GEM life, GOM and PBM have a shorter lifetime [8,9] and can be deposited on the ground (by wet and dry deposition) and dissolved in water [10]. Hence, it usually enters the environment near its source. Thus, controlling gaseous Hg(II) and Hg(0) emissions may have local and global benefits, respectively. Finally, all the mercury species dissolved in water will be subjected to oxidation or reduction reactions [11]. Thus the behavior of mercury in the environment looks complicated: mercury deposited as Hg(II) can come back to the atmosphere as Hg(0). 

Additionally, Hg(0) can react (oxidize) to form Hg(II) in the atmosphere, and Hg(II) can then reduce back to Hg(0). In other words, mercury can continuously change its form. 

Correlating Hg concentration and its forms to the sources of emission is of paramount importance to delineate its transport and fate in the environment in order to identify and, eventually, reduce anthropogenic sources. The adoption of both advanced sensors [12,13,14] and analytical devices [15,16,17] in monitoring mercury and its forms on a global scale is one of the key requirements within international programs devoted to the earth observation. Indeed, mercury has been regulated by international agreements, national agencies and recently on a global scale through the United Nations Environmental Program (UNEP)—Minamata Convention [18,19,20,21]. Since 2010, to create an international network capable of providing accurate measurements of Hg on a global scale, a European program called the Global Mercury Observation System (GMOS) has been financed [22] with the dual aim to protect the ecosystems and human health from anthropogenic emissions and to control and measure the release of mercury species in the air. 

Passive air sampling is an additional common strategy used to collect and measure gaseous Hg. Even though passive air samplers (PASs) are not real-time monitoring systems, they overcome the limitations of other approaches like active monitoring systems, since they enable the simultaneous spatial Hg measurements in different areas, thus creating a map of the Hg concentration surrounding the sources of emission [23]. PASs are widely used for environmental monitoring of mercury and other pollutants in the air. The features of passive sampling are related to the principles of green analytical procedures, typically characterized by decreasing sample treatment steps and energy-saving and in situ measurements [24]. On the contrary, a typical drawback of PASs is the coarser temporal resolution consequent to the extended exposure time required to collect a detectable amount of mercury that generally spans from weeks to months. Furthermore, the uptake profiles and sampling rate (SR) of the adsorbed chemicals need to be determined from previous calibration studies, where analyte concentrations collected on the passive sampler are compared with concentrations detected by active sampling methods, where the sampled volume is known [25].

Usually, the adsorbing material in a PAS is located inside a container protected by a membrane on a disk-like geometry or column geometry surrounded by a cylindrical diffusive barrier. The latter samplers, Radiello^®^, are the most known commercial radial-type tools that are characterized by higher adsorbing surface resulting in an increased sampling rate (SR) [26]; the con is that the uptake depends on the wind conditions, thus reducing data reliability of outdoor monitoring [27]. McLagan et al. overcame this limitation by designing a PAS constituted of a Radiello^®^ sampler placed into a protective shield for outdoor deployment [28]. Differently, in the case of axial-type PASs, pollutants diffuse along the container path until reaching the adsorbing material (tube-type passive). The SR of axial-type samplers can be set by adjusting the length of the diffusive path inside the container. Therefore, for indoor deployment (no wind), short diffusive paths are more suitable than long paths, which are used for outdoor exposure.

Activated carbon and sulfur-impregnated carbon [22,28,29,30,31] are the most common and commercialized type of adsorbing material for TGM due to the low cost and the relatively low amount of material used (≈1 g). However, since the analytical method to measure the adsorbed mercury is digestion, these adsorbing materials are disposable. Samplers based on gold materials in thin films [12,31,32] or nanoparticles (NPs) [33,34,35,36,37] have been developed and used in both active [12,38] and passive mercury samplers [31,33,35,36,37] for TGM monitoring. The high affinity between mercury and gold enables the uptake of little concentrations of mercury species upon amalgam formation. Such a mercury collection method has the advantage of being analyzed by desorption instead of by digestion, thus reducing potential sources of contamination and errors [32] and permitting the recovery of the adsorbing material upon thermal desorption and then its reusability. Therefore, even though noble metal-based adsorbing materials are expensive, their reusability makes these samplers more cost-effective and reduces the costs of measurements [39].

According to the type of material used, the gold-based samplers can collect a particular type of mercury species selectively or can be used for different applications in combination with other membranes. For instance, ion-exchange membranes (like polysulfone) [40,41] are capable of capturing selectively only GOM so that when PASs for TGM are used in combination with these ion-exchange membranes, only GEM can be quantified. Recent advances in nanotechnologies have allowed the production of a variety of highly sensitive nanomaterials with more intriguing properties than the commercial tools [42,43,44]. Nanostructured materials with unique optical, chemical, magnetic and electronic properties have been designed to improve detection. In particular, the nanostructures employing noble metals have been exploited for their strong surface plasmonic effects, color and current changes. James et al., (2012) [43] reported a sensitive and reusable Hg^0^ monitoring device based on gold nanoparticles with a limit of detection of approximately 90 ppb in air. McNicholas et al. (2011) [44] reported a very sensitive Hg^0^ system based in gold nanoparticles deposited on carbon nanotubes with a detection limit of 2 ppb in air. Santos et al. (2017) [34] used a gold NP-impregnated glass disk as a portable device for measuring the worker exposure to highly polluted environments (artisanal gold mining) by exploiting the color change induced by GEM adsorption. Indeed, mercury is also released in the air by several other human activities, such as dentist offices and fluorescence lamp factories, so that daily passive samplers or sensors should be used to monitor the workers’ exposure to mercury and prevent the associated health consequences. Therefore, the environmental monitoring and occupational health evaluation would require that sensitive mercury samplers were cheap, rapid and wearable. Gold nanoparticles decorating nanofibers of TiO_2_ have been designed to adsorb and reveal mercury from the air up to between 6 and 2 ppt_vol_ [45]. Salcedo et al. (2018) developed a device based on a cuprous iodide/polystyrene composite for mercury, exhibiting reddish color in the presence of Hg^0^ [46]. This device was able to work from 61 to 270 μg/m^3^ (high polluted), and the detection was performed by an image processing program utilizing a smartphone picture of the sampler.

Previously, Macagnano et al., (2018), designed a simple-to-prepare and -use, low-cost and re-deployable passive axial air sampler (PAS) based on a film of titania NPs (about 100 μm thick) finely decorated with gold NPs (Au@TiO_2_ NPs) [35]. The PAS was also supported by a fibrous quartz filter disk suitable for thermal desorption. This type of PAS was tested in controlled lab conditions at different parameters (temperature, relative humidity and mercury concentration) in order to assess the performance of the adsorbing material. The Au@TiO_2_ NP film showed an absorption efficiency of ≈95%, no high augmentation of uncertainty due to temperature and relative humidity, and reusability in a controlled environment. Therefore, the nanostructured and heterogeneous film was confirmed to be quite efficient in capturing and storing mercury vapor. However, such a thick layer, due to disc handling for the following thermal desorption, likely underwent microfractures, thus changing its surface-related properties. In the present study, we investigated the effects of layer thickness and arrangement on the mercury diffusion and sampling rate values when the PASs were exposed to a low polluted site and for different time. Therefore, samplers containing mercury adsorbing layers and with Au@TiO_2_ NPs were fabricated and housed in customized holders and then underwent a series of thermal cycles for mercury desorption. PASs efficiency after each thermal treatment was also assessed.

## 2. Materials and Method

All chemicals were purchased from Sigma-Aldrich (Merck KGaA, Milan Italy) and used without further purification: polyvinylpyrrolidone (PVP, 1,300,000 g/mol), titanium (IV) oxide particles (TiO_2_ anatase, ≤25 nm diameter, CAS 1317-70-0) and gold (III) chloride hydrate (HAuCl_4_, 99.999%). Ultrapure water (5.5 × 10^−8^ S/cm) was produced by MilliQ-EMD Millipore. Fibrous quartz filters (Whatman^TM^, Little Chalfont, UK) 400 µm thick with ≈2 µm pore diameter dimension and ≤3 µm fiber width were used as scaffolding and cut in 20 mm diameter disks.

### 2.1. Preparation of Au@TiO_2_ NP Dispersion

The TiO2 particle gold decoration procedure was previously described by authors [35]. Briefly, a water dispersion of TiO_2_ particles (3.71 mg/mL) containing 11.0 mg/mL of PVP (capping agent) and 1.10 mg/mL of HAuCl_4_ was exposed to UV light for 1 h (GR.E. 500 W Helios Italquartz lamp). After exposure, the color turned from light yellow to dark purple. TiO_2_ NPs were used to exploit their photocatalytic activity to reduce HAuCl_4_ and obtain gold nanoparticles anchored to TiO_2_ (Au@TiO_2_) without any additional linkage.

Au@TiO_2_ NP dispersion was centrifuged at 9000 rpm for 20 min (REMI Neya-16R centrifuge); the particles sediment was sonicated in water and then centrifuged again. This procedure was replicated three times to remove most of the PVP and to achieve an Au@TiO_2_ NP water dispersion (13.3 mg/mL).

### 2.2. PAS Fabrication

**Figure 1:**
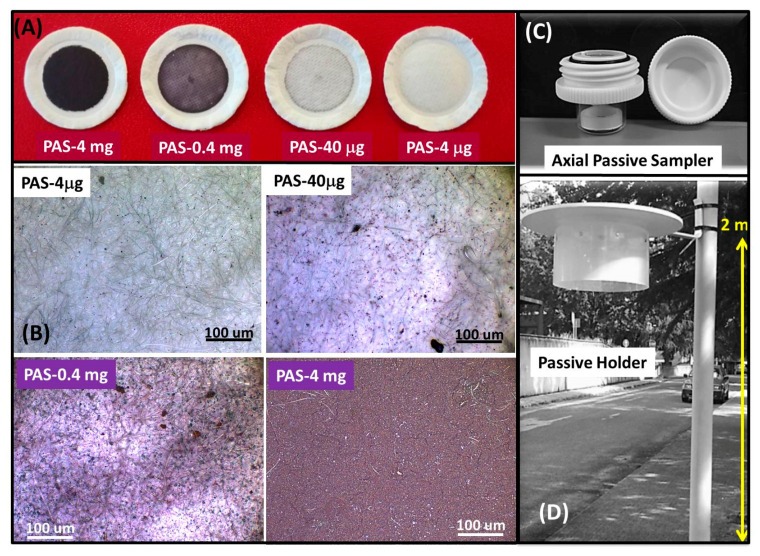
Composite picture (*right*) showing the quartz microfibrous filters upon Au@TiO_2_ nanoparticle (NP) deposition (**A**). The different colors of the discs is related to the amount of Au@TiO_2_ NPs mass-loaded. Images are captured by camera (**A**) and optical microscopy (**B**). (**C**) A passive sampler architecture showing the double screw cap and the glass vessel with the polytetrafluoroethylene (PTFE) ring inside. (**D**) The global monitoring system with the customized shelter housing up to eight passive air samplers (PASs), and anchored to a pole at about 2 m height from the ground (breathing zone).

The principles of the PASs here fabricated were the followings: The PASs worked exploiting the unassisted axial diffusion route of the gaseous mercury through a *diffusive membrane*, along a glass vessel (diffusion path), until reaching the *adsorbing membrane*. Hence, the PAS was composed of a see-through borosilicate vessel, a cap made of a nylon membrane for gas diffusion and particulate stopping, a locking ring to keep the adsorbing membrane to the vessel bottom, and finally the adsorbing membrane onto the quartz fibrous substrate (Figure 1C,D).

#### 2.2.1. Adsorbing Membrane

The adsorbing systems were based on commercial fibrous quartz filters decorated with Au@TiO_2_ NPs by drop-casting as follows: An aliquot of 300 µL of the Au@TiO_2_ NP water dispersion was poured all at once onto a 20 mm diameter fibrous quartz filter disk, using a suitable mask with 12.5 mm diameter aimed at concentrating the suspension on the filter. The mask was manually pressed onto the filter disk. This procedure provided a reproducible size of the exposure surface area and a homogenous deposition of the mass of interest over the filter. The reproducibility of the deposition method was assessed in about 120 adsorbing layers, where the deposition of the water dispersions resulted in 4.3 ± 0.3 mg of Au@TiO_2_ NPs. Some absorbent paper was placed underneath to collect the aqueous solution leaching from the quartz filter.

Similar volume solutions but with different Au@TiO_2_ NP concentrations were used in the procedure to obtain distinct masses of Au@TiO_2_ NPs deposited onto the quartz filters. By the way, the weight of the filter was measured before and after deposition of 300 µL of Au@TiO_2_ NP water dispersions to calculate the mass deposited. Specifically, different dispersions generated 4, 0.4, 0.04 and 0.004 mg of the composite layers, named as PAS4mg, PAS0.4mg, PAS40μg and PAS4μg, respectively.

The Au@TiO_2_ NP-decorated quartz scaffolds were then oven dried at 105 °C for about 24 h to remove totally the water molecules adsorbed on the filters and finally heated at 550 °C for 15 min under clean air flux to remove any trace of Hg possibly adsorbed onto the gold particles during the deposition procedure and contaminating the resulting adsorbing membranes.

#### 2.2.2. PAS Assemblage

All the components of PAS containers were dipped in an aqueous solution of HCl (0.01 N) to remove most of the adsorbed mercury and then rinsed with ultrapure water (0.055 µS/cm) in a ultrasonic bath (Branson Ultrasonic 1800, 30 min, 50 °C) and finally under ultrapure water flow. Then, they were left to dry in a glove-box under a flow of dry and pure air coming from a cylinder (5.0, Praxair) and passing through a home-made activated carbon cartridge. The assembling of the PASs occurred inside the glove-box to avoid any further contamination. The adsorbing membranes were then placed into the see-through borosilicate vessels (2.7 cm × 2.4 cm, height × diameter) and blocked to the base by polytetrafluoroethylene (PTFE) rings. The containers were then plugged with customized double screw caps (Figure 1C) (Spaziani Rolando S.r.l.), where the top cap (4.1 cm × 1.4 cm) closed and sealed the PAS to prevent Hg diffusion into the vessel during PAS storage and transport. The bottom cap (4.1 cm × 1.7 cm), instead, was sealed on the top with the diffusive nylon membrane (10 × 10^3^ μm^2^ each pore area), enabling both gas diffusion and dust particle stopping during PAS exposure to the atmosphere for mercury monitoring.

#### 2.2.3. Monitoring System Arrangement

To generate operative monitoring systems, customized shelters were fabricated to host the PASs and protect them from environmental (sunlight, heating, dust and rain) disturbances and interferences. Each shelter consisted of a top opaque rigid plastic plate (r = 15 cm) covering a ≈ 10 cm high central cylinder (r = 9.5 cm) of PP (polypropylene) to shield the PASs hosted internally and prevent possible sidewise weather interferences during Hg sampling. The plastic shelters were fabricated to host up to eight PASs, each arranged in a circle and held by clamps (Figure 1D).

Before starting the measurements, the various PASs loaded with distinct Au@TiO_2_ NP masses, then with a different surface density, were housed in the shelters. Briefly, the top cap of the assembled PASs were removed, and the opened PASs were fixed upside down in the shelter, i.e., with the diffusive membrane, exposed to the atmosphere, following a randomly distribution according to a 5:3 ratio between Au@TiO_2_ NP-loaded filters and controls (blanks, i.e., sealed PASs). PASs with different Au@TiO_2_ NP surface density were mounted in each shelter. Five replicates per each surface density of Au@TiO_2_ NPs deposited (n = 20) and 12 control samples (n = 12) were tested according to the experimental plan. Each PAS-loaded shelter was then mounted on a pole 2 m high (breathing zone). At the same time, a number of PAS4mg (n = 96) were housed in 12 shelters and mounted onto 4 different poles (in groups of 3) (Figures S5 and S6), 36 of which used as Blanks and tested over 1 year.

### 2.3. Measurements and Analysis

A double experimental approach was carried out: the first one to investigate the relationship between the layer density and the passive features; the second one to test the passive features over the time.

#### 2.3.1. Measurements

All the passive samplers were used for different periods from 1 to 200 days within 1 year to test the features of the PASs with diverse adsorbing capacities when exposed to the air for various durations (daily, weekly, monthly until half-yearly). At the end of each monitoring period, the PASs were immediately plugged and sealed with the top cap and transferred to the laboratory. The monitoring site was the campus of the CNR Research Area (Rome) (42.11, 12.64 and 45 m for latitude, longitude and elevation coordinates, respectively).

#### 2.3.2. Resampling

PASs were also assessed for their effectiveness in Hg resampling in trials consisting in 35 cycles of sorption-desorption. Briefly, a separate set of three PASs loading the same mass of Au@TiO_2_ NPs (PAS-4mg, specifically) underwent 35 cycles of outdoor exposure for periods ranging between 1–8 days followed by thermal desorption. Their results were compared to the Tekran^®^ estimated values.

#### 2.3.3. Analyses

At the time of Hg measurements, the mercury adsorbed onto the membranes was measured by thermal desorption and Atomic Spectroscopy according to the EPA Method 7473 (SW-846) [47] as follows. Each double screw cap was unplugged; the PTFE ring was removed; and the Au@TiO_2_ NP-loaded filter disc was withdrawn from the vessel, placed in a hermetic quartz crucible and heated in the oven (Forni De Marco, Rome, Italy) at ≈550 °C. During heating, pure air was used as a carrier to collect and inject the Hg^0^ vapors generated in the quartz crucible to the mercury analyzer (Tekran^®^ 2537A, CVAFS, Toronto, Canada) in order to measure the amount of adsorbed gaseous mercury. Each desorbed and restored quartz membrane was placed inside another clean and dry PAS where it was stored; then, it was ready to be reused for further measurements.

Environmental parameters, like temperature and relative humidity, were provided by a HMP7-Vaisala probe placed outdoors and close to the measurement area, while mercury concentration (TGM) was measured by Tekran^®^ 2537A. Data were collected and analyzed by Origin^®^ 2018 (Ver. 95E).

Based on the Hg values obtained through the measurements carried out, the rate of adsorption during the exposure time was calculated as the amount of Hg adsorbed per unit of time and expressed as ng/day.

The sampling rate, indeed, was calculated based on the assumption that the slope of the linear regression model can be related to the empirical value of the sampling rate according to the Equation (1) [48]:

(1) $$SR_{LinReg}=m_{Hg}/\left(C\cdot t\right)$$

where *SR_LinRe_*_g_ is the slope of the linear regression, *m_Hg_* is the adsorbed mass of mercury, *C* the measured concentration and *t* the number of the exposure days. The sampling rate in passive analysis is a crucial parameter related to the amount of analyte collected by the sampler per unit time at constant concentration. *SR* quantifies the volume of air pollutant effectively stripped per unit of time. Theoretically, it depends on both the diffusion coefficient (Fick’s First Law) of the adsorbing compound and the geometrical parameters (2) [49]:

(2) SR=DA/L

where *D* is the coefficient of molecular diffusion (pressure and temperature dependent), *A* is the surface area collecting the analyte, and *L* is the diffusive path length.

### 2.4. Material Characterization

The nanostructured layers were characterized by Atomic Force Microscopy (AFM, Flex-AFM, Liestal, Switzerland), which captured images of the layer surface in tapping mode using 190Al-G tips, 190 kHz, 48 N/m. The roughness of the PAS4mg layer was measured using SPIP 6.7.6 software (Image Metrology, Hørsholm, Denmark) over image areas of 100 µm^2^ (10 × 10 µm). A plane correction process was performed on all of the topographical images. The roughness parameter here reported within the defined area was the roughness average (i.e., the difference in the height of each point compared to the arithmetical mean of the surface) (Sa) measured both over the whole surfaces and each micrograin (up to 50), respectively. SEM (Scanning Electron Microscope Jeol JSM 6010LA) micrographs were also taken with and without a gold sputter coating in a Balzers MED010 unit. High-Resolution Transmission Electron Microscopy (HR-TEM) analysis of the Au@TiO_2_ NP powder was performed by a ZEISS Libra 200FE microscope (Oberkochen, Germany). The size distributions were manually calculated, counting more than 400 NPs by iTEM software (Olympus SIS, Muenster, Germany).

Optical micrographs were captured by DMC4500 (5 MPx) camera mounted on the optical microscope (Leica DM2700M) for the quality evaluation of the nanocomposite filter layer coverage.

## 3. Results and Discussion

The doping of titania (TiO_2_) with nano-gold decorations has recently enabled the development of highly efficient materials for sensors, energy, smart coating and environment [50,51,52,53,54]. In the present study, the authors selected one of the simplest, cost-effective and green procedures [35]: AuNP-decorating TiO2 were synthesized by reducing HAuCl_4_ onto titania particles in aqueous suspension by a UV-light-catalyzed reaction. Hence, no sophisticated technologies or harsh solvents were used. This process caused a significant increase in the adsorbing surface exposed to atmospheric mercury. Among the thermally resistant substrates, fibrous quartz was selected to keep porous the film deposited by drop-casting and preventing its excessive packing, thus both facilitating the mercury adsorption and the following thermal desorption during the passive membrane regeneration.

### 3.1. PAS Characterization

Depending on the amount of Au@TiO_2_ NPs deposited, the filters appeared differently colored. Of course, it depended on the distribution of the nanoparticles onto the substrate. The surface density changed from 3.26 × 10^−2^ mg/mm^2^ to 3.26 × 10^−5^ mg/mm^2^ when the loaded mass passed from 4 mg to 4 μg. Figure 1 presents a photograph of the four quartz filters coated with the different Au@TiO_2_ NP loadings (Figure 1A) and their optical microscope pictures (Figure 1B). Both groups of images show that at the lowest concentration (PAS4μg), the substrate looks white (that is the pristine color of the filter). In the optical microscope pictures, the original white fibers showed some purple aggregates (Au@TiO_2_ NPs) appearing as tiny spots in the matrix that increased in number by increasing the mass of Au@TiO_2_ NPs deposited, causing the intensity of the purple color to also increase.

**Figure 2:**
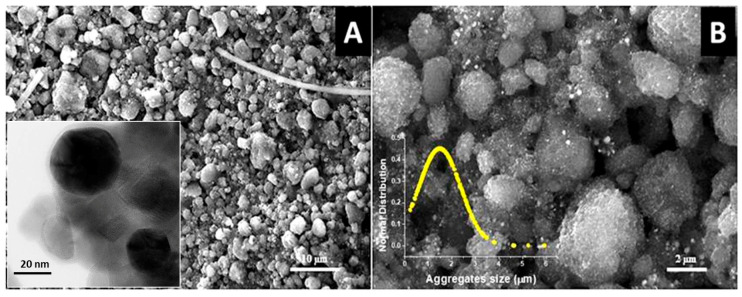
SEM micrographs of PAS4mg at 1700× (**A**) and 7000× (**B**) magnification showing an almost complete coverage of the quartz fibers with Au@TiO_2_ NPs and micrometric aggregates on the top. Inset (**A**) shows a HR-TEM micrograph of the powder; inset (**B**) depicts the normal distribution of the diameter size of the microaggregates.

Optical microscope pictures highlighted that the complete coverage of the fibrous matrix was achieved when a 4 mg Au@TiO_2_ NP mass was loaded (Figure 1B), i.e., when the surface density was at least 3.26 × 10^−2^ mg/mm^2^. The complete coverage was confirmed by SEM analysis. The top view of the surface of the fibrous quartz, captured by SEM upon drop deposition of 4 mg Au@TiO_2_-NPs and thermal treatments (PAS-4mg), showed that the filter was coated by the nanocomposite film (Figure 2A), whereas the Au@TiO_2_ NPs generated aggregates that induced an extremely rough and wrinkled film surface. The plot of the normal distribution (Gauss curve) of the aggregate size indicated that the mean diameter was 1.49 ± 0.88 μm (Figure 2B inset).

A higher magnification of the surface pointed out the presence at the film surface of pillars, valleys, canyons as well as some tiny voids between the Au@TiO_2_ NP aggregates facilitating the gas diffusion through the PAS (Figure 2B). In addition, the nanoparticle grains showed a rough surface maybe caused by the peculiar architecture of the composite nanoparticles. The nanocomposite material was previously characterized by authors [35].

Figure S1A–C and the inset picture in Figure 2A (an HR-TEM micrograph) confirmed the presence of highly crystalline and globous-shaped gold nanoparticles (a mean of about 32.6 nm) in close contact with the anatase crystalline support (AuNPs appear slightly darker with respect to the anatase support).

**Figure 3:**
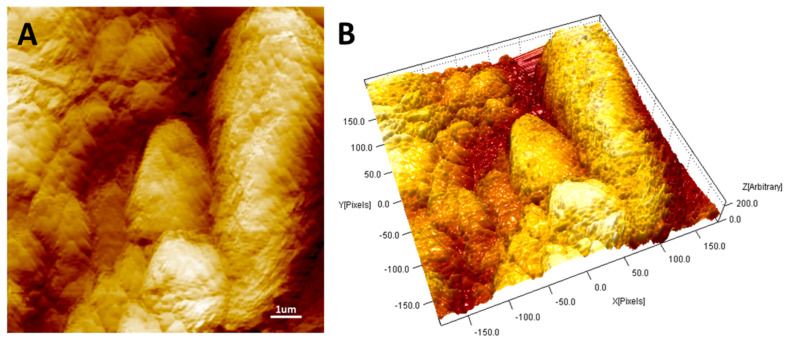
(**A**) Two-dimensional and (**B**) three-dimensional PAS4mg Atomic Force Microscopy (AFM) micrographs (10 × 10 μm surface area).

Then, the nature and the final architecture of the nanocomposite layer looked responsible of the high roughness of the material and then of the high contact surface. AFM analysis (2D- and 3D-map) of a 10 × 10 μm surface topography of the PAS4mg surface confirmed the presence of a nanoroughness due to the arrangement of the microaggregates in the deposited film distributed throughout the surface (Ra ≈ 107 ± 25 nm) (Figure 3A,B, respectively).

The similar analysis performed on a smaller area focused on single microaggregates proved the presence of a nanoroughness even on each micrograin (Ra ≈ 11 ± 6 nm, average roughness) previously observed also in the SEM picture of Figure 2B, whereas each micrograin appeared exceptionally wrinkled. However, in the AFM micrographs, the Au@TiO_2_ NP grains also appeared densely packed, although providing some spaces between the particles (darker areas).

A comparable result had already been observed by the authors on the surface of a thicker layer (10 mg mass) of the same material dropped on the same substrate [35], confirming that such a microgranular surface arrangement was not dependent on the thickness but on the preparation procedures and the material properties.

**Figure 4:**
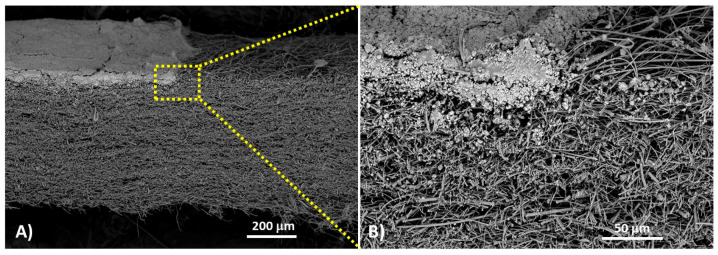
SEM micrographs (backscattered electron composition image—BEI, 10 kV, no gold sputtered, 90×) of a cross-section of a PAS4mg along the edge of the Au@TiO_2_ layer showing the layer arrangement on top of the quartz filter (**A**); a magnification of the particle distribution under the top layer (430×) (**B**).

Looking at the cross-section of a PAS-4mg substrate, the one most loaded (Figure 4A), that is, the aggregates of Au@TiO_2_ NPs in the deposition zone, appeared distributed within 50 µm depth, with the nanocomposite clusters being more concentrated approaching the surface until forming a densely packed layer on top (Figure 4B).

However, the interface between the top layer and the fibrous substrate underneath was not defined by a definite boundary. On the contrary, the aggregates and the fibers appeared as integrated into a composite fibrous scaffold, where smaller Au@TiO_2_ NP clusters were bound to the fibers at greater depth, and larger microaggregates were observed close to the surface until forming the dense layer aforementioned (Figure 4B). Because of the peculiar architecture of the Au@TiO_2_ NP film, the PAS-4mg filter was expected to display a highly interactive surface and fast adsorption processes (as well as thermal desorption) but was also permeable enough, enabling gas diffusion.

Moreover, since the top layer was partially underpinned by the quartz microfibers, it was strong enough to be handled without undergoing damages or fractures. On the other hands, the fibrous quartz substrates coated with smaller amounts of nanoparticles involved the formation of fragmented and uneven films characterized by islands made of differently sized aggregates and adsorbed onto the fibers (Figures S2–S4).

Here, due to substrate handling, an easier detachment of the nanoparticles from fibers since they were only physically joined was expected. Therefore, PAS4mg (dS ≈ 32.6 μg/mm^2^) looked decorated with the minimum amount of material capable of ensuring the development of a thin film onto the substrate used.

### 3.2. PAS Exposure

The various PASs loaded with distinct Au@TiO_2_ NP masses were then measured in the amount of mercury adsorbed upon exposure for different periods to evaluate the rate of adsorption of the gaseous mercury and the corresponding sampling rate (SR), as well as to assess the dependence of the amount of Hg adsorbed on the mass of Au@TiO_2_ NPs loaded in the PASs and the effectiveness of the adsorbing membranes (MSs).

**Figure 5:**
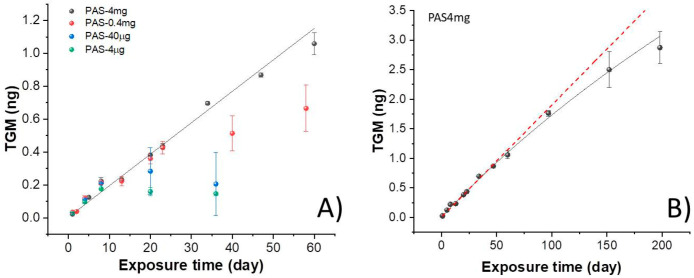
(**A**) Uptake curves (adsorbed mercury versus exposure time) of PAS4mg, PAS-0.4mg, PAS40µg and PAS4µg samples. Vertical bars correspond to standard deviation and dark gray line to linear fit of the PAS-4mg values (R^2^ = 0.995). (**B**) A non-linear fitting of PAS4mg curve within an exposure time of 198 days compared to a linear fitting (the red dotted line).

**Figure 6:**
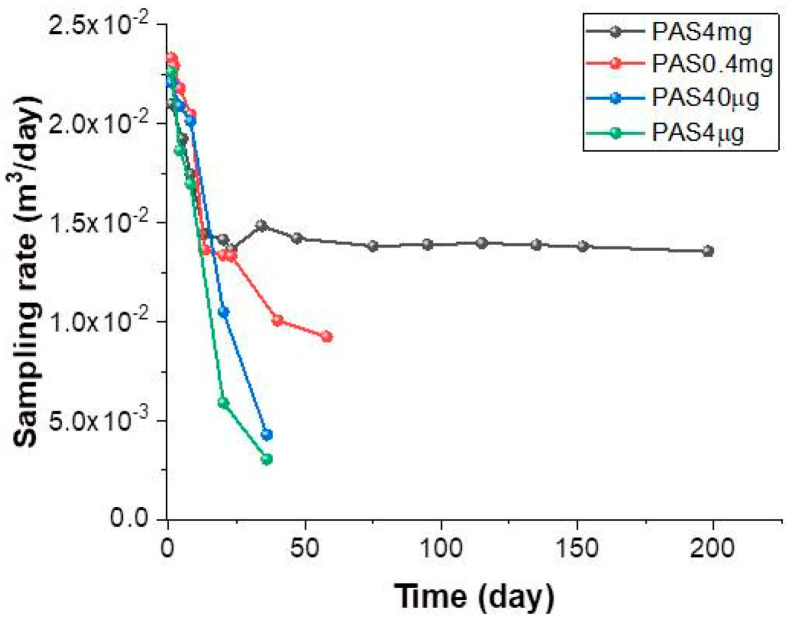
PAS sampling rate calculated for the samplers coated with different Au@TiO_2_ masses and exposed to outdoor air for increasing periods (days).

During the exposure time of the PASs, the gaseous Hg concentration was measured by Tekran^®^, which is one of the most widely adopted instrument for continuous measurements of atmospheric TGM (ng·m^−3^). In the present work, Tekran^®^ was used with the dual function of environmental monitoring system and PAS-Hg analyzer. The average daily Hg concentration in the atmosphere through the investigated period measured by Tekran^®^ was 1.24 ± 0.32 ng/m^3^, with daily and seasonal fluctuations. The analysis of mercury adsorbed on PAS as a function of the exposure time (the uptake curves of Figure 5) for all the types of PASs showed that PASs loaded with different mass of Au@TiO_2_, as well as area density, collected approximately and linearly the same amount of Hg until 8 days of exposure (Figure 5A). However, the different effects of the architecture of the layers were underlined initially in PAS40µg and PAS4µg, which appeared saturated after 7–8 days of exposure, when the gaseous mercury adsorbed per day started to decrease. Furthermore, prolonged exposure of these PASs up to 40 days caused a decrease in the total absorbed mass of mercury. Such an effect could be also due to the detachment of the adsorbing particles from the filter following the disc handling for the thermal desorption procedures. Conversely, PAS0.4mg samples, having a higher NP loading and surface density (3.26 μg/mm^2^), followed a linear trend until ≈23 days of exposure. After that, they deviated from linearity, causing the adsorbing rate to start decreasing. The gray line in Figure 5A represents the linear regression of PAS4mg values forced passing through the origin. The linear shape confirms that this amount of Au@TiO_2_ NPs loaded on PAS (4 mg) with the highest area density (32.6 μg/mm^2^) as the threshold value, in our study, is capable of providing a constant rate of mercury adsorption until about 60 days of exposure. Here, the calculation of the rate of adsorption within the first sixty days reported a value of about 18.5 ± 0.44 pg/day at a Hg^0^ average concentration in air of about 1.24 ± 0.32 ng/m^3^, which is a common concentration in low-polluted sites [28]. Therefore, ≈1.5% of the average gaseous concentration of mercury could be adsorbed daily. Such a linear behavior in mercury uptake suggests that the adsorbent layer had not approached its total adsorption capacity (saturation) for at least 60 days of exposure. However, a slight decrease in the adsorption rate occurred when these PASs was exposed for a longer period. Figure 5B depicts in more detail a non-linear fitting of the curve with an apparent saturation of the binding sites over the second half of the sampling time (more than 100 days). However, this result could depend on several reasons not entirely related to the binding sites saturation. For instance, it could be due to the seasonal variability of the mercury concentration, which decreased from summer to autumn (1.34 ± 0.44 ng/m^3^ and 1.18 ± 0.44 ng/m^3^, respectively) (confirmed later by Figure 8). On the other hand, the possible interference of other compounds in the polluted atmosphere interacting with the adsorbing surface of PASs during the prolonged exposure could also reduce the adsorption efficiency of the devices, thus preventing Hg from reaching the Au-NPs. Even chemical reactions occurring at the interface between air and the Au@TiO_2_ NP aggregate surfaces and resulting in the loss of Hg could not be excluded. For instance, ozone could remove the adsorbed Hg affecting the final result [32,55,56]. In any case, these results proved that the PAS4mg membrane was properly working within a hundred days. Taking into account the daily TGM_av_ concentration values measured by Tekran^®^, the daily sampling rates were calculated, from Equation (1), per each PAS (Figure 6).

It is known that SR is strongly dependent on the characteristics of the pollutant and the design of the sampler [57].

In the figure, high SR values (in the range between 2.0–2.3 × 10^−2^ m^3^/d for all the Au@TiO_2_ NP-loaded PASs) during the first days of exposure are depicted, followed by a sharp decrease over the first week, but with different slopes in the various PASs.

Such a decreasing trend of SR was also observed by Papa et al. (2018) in axially diffusive PASs based on gold NPs deposited onto quartz fibers and differently arranged on the surface. The SR value was confirmed to be higher the first day of exposure, then decreasing gradually during the first week until stabilizing during the following days [36]. In the present study, too, after the first week of exposure, SR remained constant (about 1.398 × 10^−2^ ± 0.051 × 10^−2^ m^3^/day) for both PAS-0.4mg and PAS-4mg until ≈30 d. In PAS4mg, SR remained apparently almost stable even for a longer period, with a maximum decrease of about 3% (1.356 × 10^−2^ ± 0.061 × 10^−2^ m^3^/day) after 198 days (Figure 6). Conversely, the 4–40 μg masses reported a constant decline in SR without ever reaching a constant value. Presumably, higher SR values might occur when the gaseous elemental mercury is adsorbed onto the outside sites of the external layer of Au@TiO_2_NPs. Once all external sites are occupied by mercury, Hg in air should diffuse through the channels within the layer reaching the inner sorption sites [58], thus increasing the diffusive path L (Equation (2))and reducing the SR value.

The concentration of the gaseous mercury upon the adsorbent material is, indeed, low at the beginning of sampling, resulting in a higher concentration gradient with a faster adsorption of the gaseous mercury. Since uptake is a cumulative process and SR is relative to the whole sampling period, the initial values can be considered irrelevant when PAS works for more than 1 week. Since the mercury stripping seems constant until 200 days, the efficiency of the PASs seems to be affected only slightly by the parameters described above and lowering Au features. Therefore, the non-linearity of the curve in Figure 5B should be largely due to the lowering of the TGM and then of the related uptake (afterwards confirmed by Figure 8). On the other hand, the dramatic SR daily drop for the less loaded PASs (Figure 6) means that, despite their sensitivity to mercury, they cannot be used for long deployments. The higher sampling rates of both PAS0.4mg and PAS40μg within the first week when compared to the PAS4mg one confirm their relationships with the surface area across which diffusion occurs, which is expected to be higher in less compact architectures. Therefore, concerning the chance of using the investigated passive samplers, the data suggest that there is a mass threshold of the described adsorbing layer and then a surface density, which necessary to make available these devices for monitoring campaigns.

Then, once an SR is calculated as a constant value for a specific kind of PAS, such a device could be used to estimate the mercury concentration in air by knowing the mass of the adsorbed analyte (i.e., the gaseous mercury), according to Equation (3):

(3) $$C\;\left(ng\cdot m^{3}\right)=\frac{adorbed\;mass_{Hg}\left(ng\right)}{deployment\;time\;\left(day\right)\cdot SR\left(m^{3}\cdot day^{-1}\right)}$$

**Figure 7:**
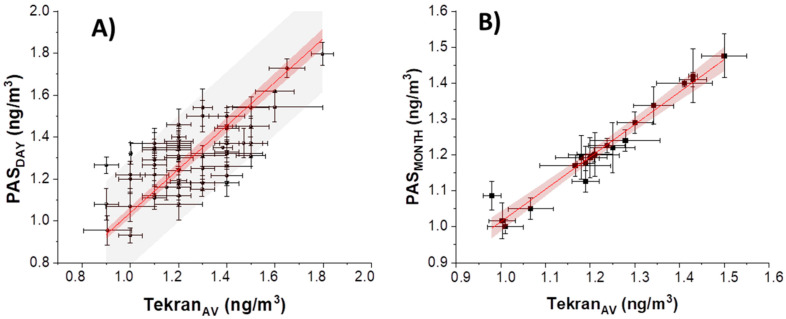
(**A**) Linear fitting of PAS data vs. Tekran collected day by day forced to pass through 0; (**B**) linear fitting of PAS data vs. Tekran collected monthly (SR: 0.014 m^3^/day) forced to pass through 0. The red band depicts a 95% confidence band; the gray band depicts 90% of prediction.

In the literature, depending on the range of environmental mercury concentration to be monitored, several PASs have been properly designed with different SR values [49]. PASs possessing low SRs are commonly used for long-term exposures or for high-polluted environment (e.g., for mapping wide areas or placed near factories, respectively) [59]. In these cases, the passive saturation is slowed down. For instance, Brown et al. developed PAS based on gold-coated silica placed in an axial tube without diffusive membrane possessing an SR of 0.00031 m^3^/day [60], where the minimum exposure time (in low concentration environment) is ≈1 year or a number of months, depending on the level of pollution. Brumbaugh et al., (2012) designed a liquid sorbent sampler containing nitric acid and gold cation surrounded by a polyethylene barrier that limits the uptake of mercury with an SR of 0.002 m^3^/day, thus, the minimum exposure time is ≈4 weeks if the atmosphere Hg concentration is higher than 2 ng/m^3^. PASs possessing high SR are used generally in an environment with a low Hg concentration or low exposure time: Hang et al. designed gold-coated quartz fibrous filters with an SR of 6.6 m^3^/day [41]. Such devices are preferred in wearable monitoring systems and are also sensitive to small variations of Hg concentrations. In the present work, only the designed PAS4mg reached a constant SR of 0.01398 m^3^/day (≈3% uncertainty, for exposure >7 days), which enables the use of them in a low-polluted environment until six months, or, eventually, a highly polluted environment (e.g., workplaces) for a shorter time. However, taking into account the fact that the PAS had higher sampling rate values within the first deployment week, it is expected to be used also for shorter-term monitoring. The minimum exposure period necessary to sorb detectable amounts of Hg was proved to be 1 day. However, when the gaseous Hg concentration values were estimated from PASs exposed within 1–8 days according to Equation (1), a fair error in concentration prediction, more specifically, an overestimation, was reported if the constant SR was used for calibrating (i.e., ≈1.398 × 10^−2^ m^3^/day). Conversely, each predicted concentration appeared closer to the instrumental data when the PASs were calibrated with the 7-day exposure SR average (i.e., 0.018 m^3^/day). In more detail, Figure 7B depicts a plot, whereas TGM concentration values from PASs working between 1 and 7 days were compared with the mean values measured over the same days by Tekran. The linear regression with intercept forced to 0 changed from 1.35 ± 0.02 ((R^2^ = 0.97) to 1.03 ± 0.02 (R^2^ = 0.97) if the SR used was 0.018 m^3^/day, thus, the data discrepancies were significantly reduced (Figure 7A,B), and the whole assessed concentrations entered within the 90% prediction band.

On the other hand, a linear correspondence between TGM concentrations from instrumental and PAS data is also depicted in Figure 7B, where measurements were related to more than 10 days of exposure (named as PAS_MONTH)_. Here, a closer relationship was observed when data obtained by Tekran were averaged and plotted against those measured through PASs, allowing us to observe overlapping between the data measured and estimated (95% prediction band).

A passive sampler can be dependent on the meteorological factors, as well as the atmospheric mercury concentration. Therefore we tried to identify their effects on the presented PASs and on the TGM levels. Guo et al., (2014) [29] as Skov et al., (2007) [29] did not find significant effects of temperature on SR despite Gustin et al. (2011) [32] and McLagan et al. (2017) [61]. Similarly, Huang et al. (2014) observed effects of %RH (relative humidity percentage) [27], while other studies did not report any effects on passive samplers [29,61]. Of course, it depended on the materials and strategies adopted to uptake mercury from air. Macagnano et al., (2018) [35], previously found a very slight relationship between the estimated mercury concentration and temperature (increasing +0.1% per Celsius degree, in a thermal range between −20 and 60 °C) or relative humidity levels (+0.06% per %RH unit, between a dry and a 70% humid environment). On the other hands, both the meteorological parameters can affect the mercury distribution among the different global ecosystems (soil, water, atmosphere). Thus, in the present study, we tried to use the passive samplers as a potential tool to monitor both atmospheric TGM over 1 year, and the effects of T and %RH on it. Therefore, the passive measurements were provided outdoors and were related to the meteorological parameters as temperature and relative humidity due to diurnal cycles and changing seasons. In the present study, wind effects were not considered since the selected area for PAS deployments was close to a building repairing the samplers against variations in atmospheric turbulence.

**Figure 8:**
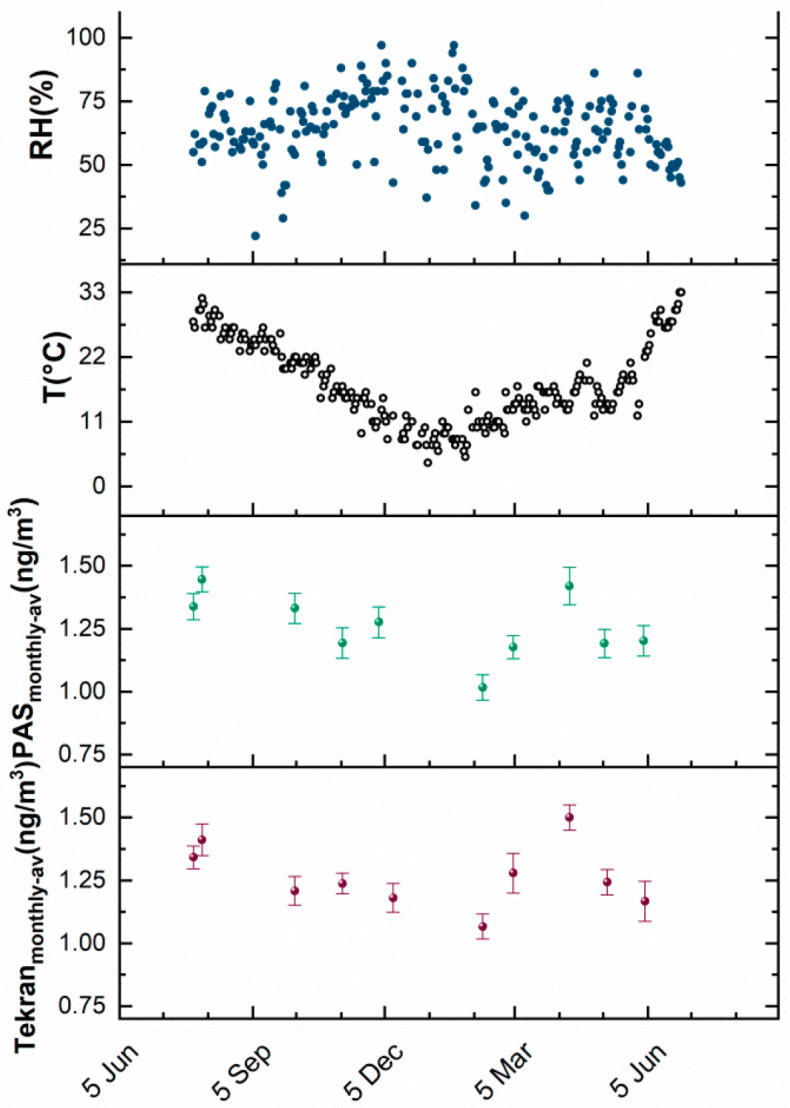
Composite plot depicting the daily trend of %RH (**●**) and T (o) over the sampling time (1 year); averaged data from 1-month deployed PAS (**●**) and instrumental data (**●**) recorded within the same sampling time. The latter were averaged (Tekran_monthly_) to be compared to PAS values (PAS_monthly_).

However, the opaque shield of the customized shelter (Figure 1D) protruding about 5 cm from the PAS entry was aimed at lowering the wind contribution; then, it was expected to be negligible. During the Hg monitoring campaign, RH (%), T (°C) and TGM concentrations were measured and reported as a function of the exposure period (Figure 8). During the seasons, the temperature mean values changed almost linearly from +33 °C to +2 °C and back to +35 °C (from July to January, to the next July, respectively) (Figure 8); conversely relative humidity mean values changed from 20 to 95% (rainfalls) without definite trends related to the seasons. Looking at the concentration of mercury in the air, as expected, it was higher in the warm months [62].

**Figure 9:**
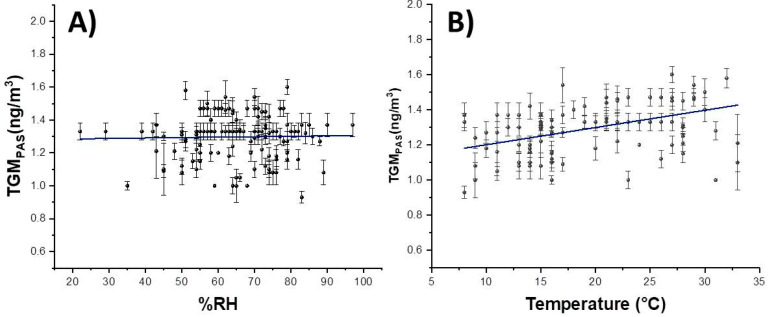
Linear fittings of collected data vs. %RH (**A**) and T (**B**) values.

Furthermore, PAS and Tekran^®^ data seemed to report substantially similar trends and value ranges (Figure 8). Figure 9 depicts the assessed concentration of gaseous mercury versus the relative humidity (A) and temperature (B) measured over the sampling period.

Each plotted value is the result of the mean of five samples working over the same days and at a defined meteorological condition. A slight relationship between mercury concentration in the air and temperature (linear regression slope of about 6.63 ± 4.46 pg/m^3^·deg; intercept: 1.149 ± 0.1256 ng/m^3^; R^2^: 0.013). Taking into account the mean value of the gaseous concentration mercury, such a slope seems to be slightly higher (≈+0.6% per Celsius degree) than the rate measured in the lab (+0.1% per Celsius degree) [35] and only due to the different interactions between the material and the mercury at different temperature values.

Such a positive correlation is in agreement with the literature, as it had been proved that solar radiation affects the flux of mercury from the soil [62,63,64].

Conversely, no significant linear relationships with humidity could be observed.

### 3.3. Resampling

The ability of gold to desorb mercury at ≈500 °C could allow, in principle, the reusing of the adsorbing material for more measurements. Although this capacity is well known in the literature, the PAS-based resampling was conducted in only a few studies [35,36,37,38,43].

Moreover, it is known that noble metal-based PASs can undergo memory effects [49,50,51,52] and lose their structural integrity (loss of adsorbing material) [65], which can influence sampler accuracy [49].

**Figure 10:**
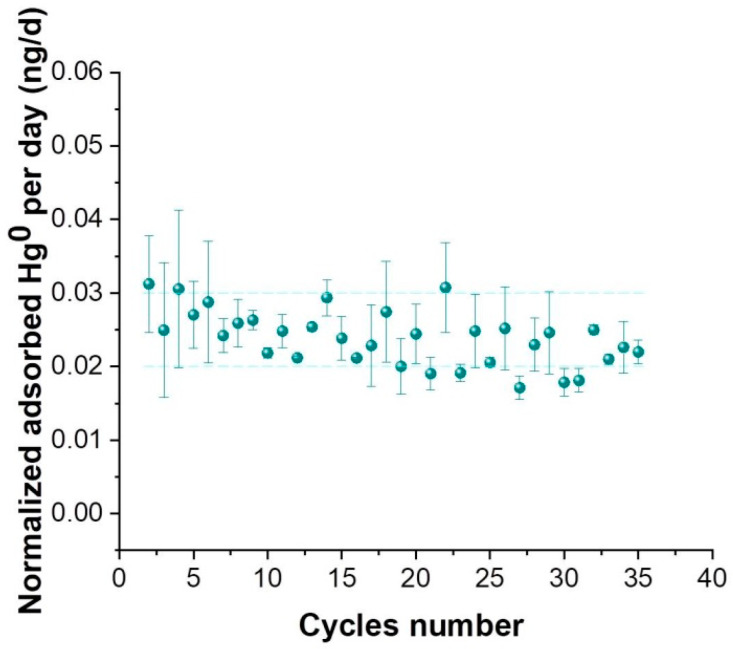
Normalized adsorbed mercury data as function of exposure cycles with an error bar for standard deviation.

Hence, PAS-4mgs were tested to verify the possibility of Hg resampling with the PASs here proposed. The weighted average of the normalized adsorbed mercury was 23 ± 5 pg/day, compatible with the daily values adsorbed by newly prepared membranes (Figure 10).

On the other hand, the linearity of the uptake curve previously described (Figure 5) combined with the capacity of PAS to be reused in Hg resampling cycles (Figure 10), seemed to confirm the high stability of the adsorbing membranes here fabricated and used to create PASs.

## 4. Conclusions

Exploiting both the strong affinity between gold and mercury and the high surface/volume ratio, differently mass-loaded films, made of TiO_2_NP photo-decorated with AuNPs, were easily deposited by drop-casting onto a microfibrous filter of quartz and then investigated as promising adsorbing structures for newly designed passive samplers for gaseous mercury monitoring. The fibrous structure could hold and group the nanoparticles in microclusters over and among fibers but achieved a quite compact and stable coverage only when a 4 mg mass was used, with a 32.6 μg/mm^2^ surface density. The high roughness as the high porosity, related to the irregular edges of the aggregates, improved both the adsorbing surface and the gas diffusion, affecting the mercury adsorption process. These discs, properly housed in borosilicate vessels and provided with a protective nylon membrane, were investigated as promising monitoring systems for outdoor mercury pollution in a low-contaminated site (C_av_ ≈ 1.24 ± 0.32 ng/m^3^) for up to six months. All the measurements were carried out in parallel with an analytical instrument for mercury detection (Tekran^®^) and with the dual function of studying the characteristics of the material for passive sampling and validating, as a preliminary, the PAS features in the measuring campaign. The quality of the composite layer deeply affected the rate of TGM adsorption values that declined quickly enough over time and proportionally to the mass-loaded PASs, thus suggesting a gradual saturation of the binding sites. Over time, Au@TiO_2_ NPs were detached from filter when the coverage was not arranged in a continuous film, presumably due to weak adhesiveness of the nanoparticles to the silica scaffold. On the other hand, PAS4mg reported a linear rate of adsorption up to a hundred days, confirming the greatest stability over time as capacity of adsorption. The samplers loaded with a smaller amount of nanoparticles (comprised between 4–40 μg masses) reported a constant decline over time in the SR values without ever reaching the equilibrium, except for PAS4mg which remained stable over a longer time. Intermediate performances were observed in PAS0.4mg. These results, related to the different film framework, enhanced the need to design, for long-term monitoring systems, PASs based on Au@TiO_2_ nano-assembled membranes with a compact and stable architecture. In fact, PAS4mg systems worked cyclically for 1 year with continuous deployments for up to six months, providing data comparable to those achieved from an analytical instrument. The high sensitivity of the membrane also enabled the obtention of readable values from daily TGM measurements, but taking into account a proper calibration of the passive samplers in order to avoid data overestimation. Finally, the samplers were tested following 35 cycles of thermal restoration, thus confirming the keeping of their features as well as their more sustainable use. On the other hand, although the manufacturing process was reproducible, a greater control on the gold size is desired to obtain a more uniform nanocomposite architecture. Finally, all the collected data were related to a confined site, low polluted and sheltered from the wind and not subjected to the effects of salt and fine sands as well as wider thermal excursions. Each of them could considerably affect, in different ways, the features of the device. Therefore, further investigations are in progress in environments that are more polluted and in the presence of potential interferents, such as chlorides and sulfides, more windy and in extreme environmental conditions, and finally covering larger monitoring areas in order to be promoted as potential devices for global monitoring campaigns.

## Supplementary Materials

The following are available online at https://www.mdpi.com/1424-8220/20/21/6021/s1, Figure S1: TEM/STEM/HRTEM TiO_2_@Au NPs powder characterization, Figure S2: SEM micrographs of PAS0.4mg, side views; Figure S3: SEM micrographs of PAS40μg, side views; Figure S4: SEM micrographs of PAS4μg, side views Figure S5: passive assembly procedures; Figure S6: PASs deployment.
