# Supplementary material for: Characteristics and Performances of a Nanostructured Material for Passive Samplers of Gaseous Hg
Source: Sensors (Basel). 2020 Oct 23;20(21):6021. doi: 10.3390/s20216021 (PMC7660345; doi:10.3390/s20216021)
Supplement: Supplementary file 1 [file sensors-20-06021-s001.pdf]

SENSORS (MDPI)

Special Issue: ***Global Mercury Assessment Sensing Strategies***

SUPPLEMENTARY MATERIAL in

Characteristics and performances of a nanostructured material for passive samplers of gaseous Hg

J. Avossa[1,3], F. De Cesare[2,3], P. Papa[3], E. Zampetti[3], A. Bearzotti[3], M. Marelli[3], N. Pirrone[3], A. Macagnano[3,2]

S.1. Composite nanostructure characterization

TiO2@AuNPs powder samples for transmission electron microscopy (TEM) measurements were slightly grounded in an agate mortar, then suspended in isopropyl alcohol, sonicated for 10 minutes, and dropped onto a holey-carbon coated copper TEM grid. TEM and scanning transmission electron microscopy (STEM) analysis were performed by a ZEISS Libra 200FE microscope (Oberkochen, Germany) after complete solvent evaporation in air. The size distribution was assessed counting more than 400 NPs by iTEM software (Olympus SIS, Muenster, Germany).

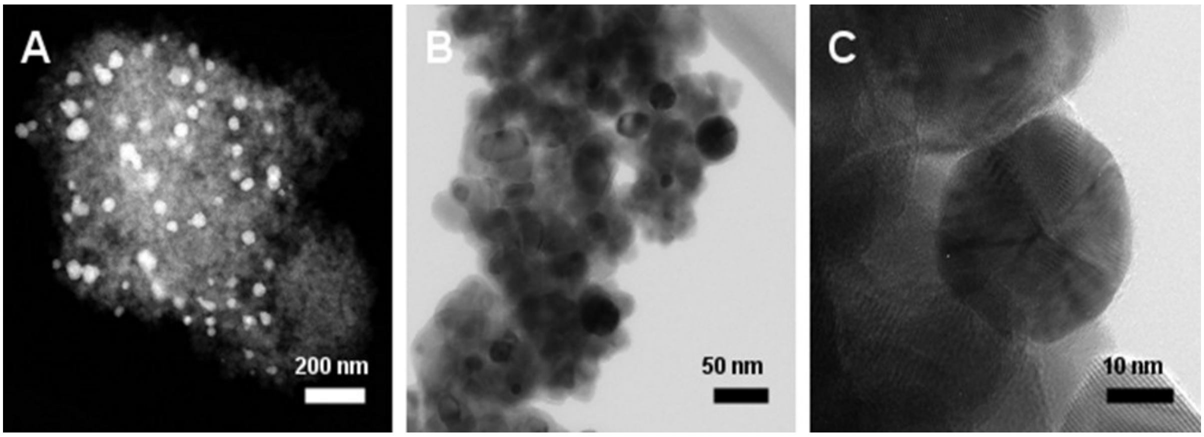

Figure S.1. Representative STEM (A), TEM (B) and HR-TEM (C) micrographs of Au@TiO_2 powder.

The composite Figure S.1.A depicts a low-magnification HAADF-STEM micrograph, where high-angle annular dark field scanning transmission electron microscopy highlights a good dispersion of gold nanoparticles onto the TiO_2 support. The gold NPs, assessed about 20-40 nm, pop-up brighter from the grayish support thanks to the STEM Z-contrast in the dark field mode. Figure S.1.B shows a micrograph acquired in the bright field TEM mode, enhancing darker and well-shaped AuNPs close to the TiO_2 grains with comparable sizes and morphology. Lastly, HR-TEM micrograph (high resolution TEM, Fig.S.1.C) confirms the crystalline structure of the well-shaped and faced AuNPs as the TiO_2 grains. More information about the characterization of the material are provided in Ref. 35 of the

manuscript (Macagnano, A.; Papa, P.; Avossa, J.; Perri, V.; Marelli, M.; Sprovieri, F.; Zampetti, E.; De Cesare, F.; Bearzotti, A.; Pirrone, N. Passive sampling of gaseous elemental mercury based on a composite tio2np/aunp layer. *Nanomaterials* **2018**, *8*, 798, doi:10.3390/nano8100798)

SEM (Scanning Electron Microscope Jeol JSM 6010LA) micrographs of the more diluted passive samples were taken without a gold sputter and at different magnification. The increasing dilutions of the initial PAS suspension (13.3 mg/mL) involved a significant change in the morphology and structure of the adsorbent film. Here, the partial, clustered and fragmented coverage of the fibrous substrates are reported (Figures S.2.A,B). The SEM micrographs, detecting the backscattered electrons, highlighted the different chemical composition of the material. In fact, since gold has a higher atomic number than the other elements (i.e. Ti and O) , it backscattered electrons more strongly than the other ones, thus appeared brighter in the image. Thus bright spots emerged from the microparticulate, confirming the different chemical composition of the material.

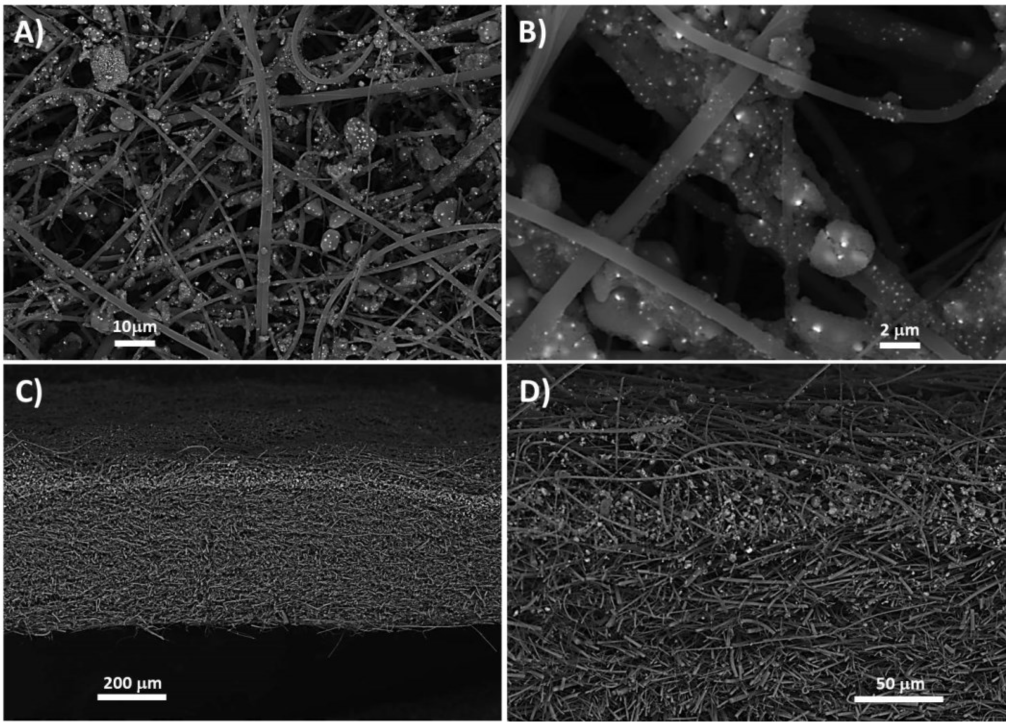

Figure S.2. SEM micrographs (BEI 20kV) of PAS0.4mg at 1000X (A) and 5500X (B) magnification, showing the quality of the coverage of the fibrous substrate with Au@TiO_2 NPs (C:1.33mg/mL); a cross section along the edge of the Au@TiO2 layer showed the "presumed layer" arrangement for adsorbing gaseous mercury (90X) on top of the quartz filter (C) and just under the surface (430X)(D)

The distribution of the TiO_2@AuNPs in PAS 0.4mg resulted very fragmented, with the composite material aggregated substantially along the fibers to which seemed to adhere. Such an arrangement was due to the low surface density achieved (d_S=3.26 mg/mm^2) which prevented particles from aggregating into a more compact layer. The membrane side view (Figure S.2.C,D) seems to suggest a distribution of the nanocomposite clusters of about 40 mm inside the fibrous scaffold, being more concentrated approaching the surface, but never forming a packed layer on top. As a consequence, a further dilution of the

TiO$_2$@AuNPs suspension resulted in a more spaced distribution of the particles (Figure S.3.A,B) over the fibrous substrate (PAS40μg). Furthermore, it seems hard to quantify the depth reached by the particles because no organized architecture is noticeable (Figure S.3.C,D).

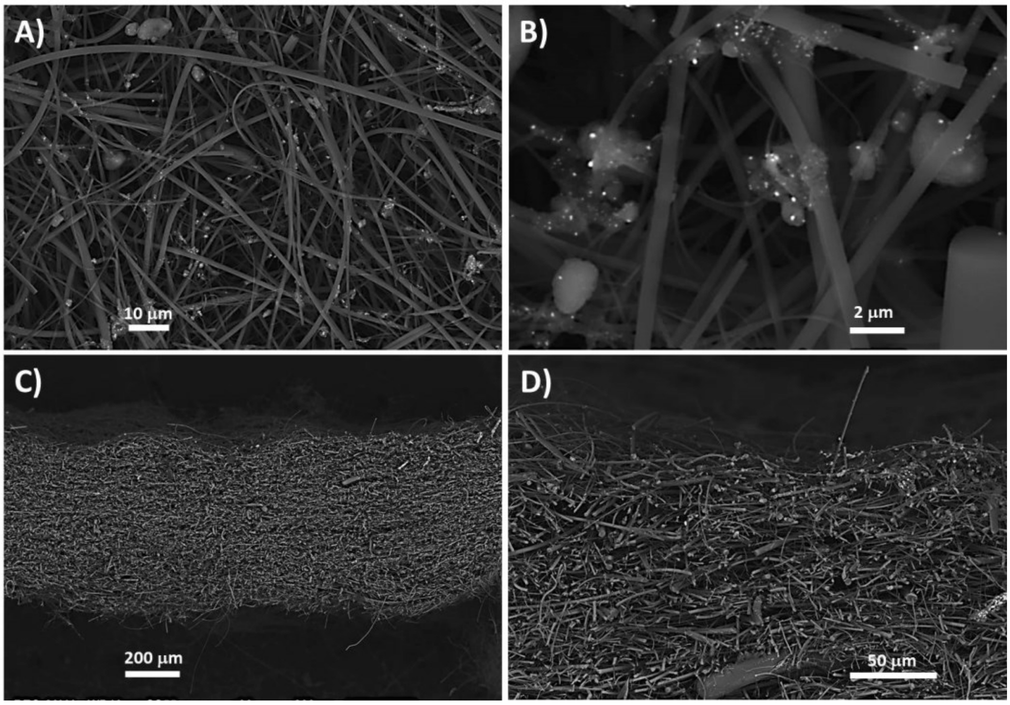

Figure S.3. SEM micrographs (BEI, 20kV) of PAS40μg at 1000X (A) and 7000X (B) magnification showing the quality of the coverage of the fibrous substrate with Au@TiO$_2$ NPs and side views magnified 90X (C) and 430X(D), respectively

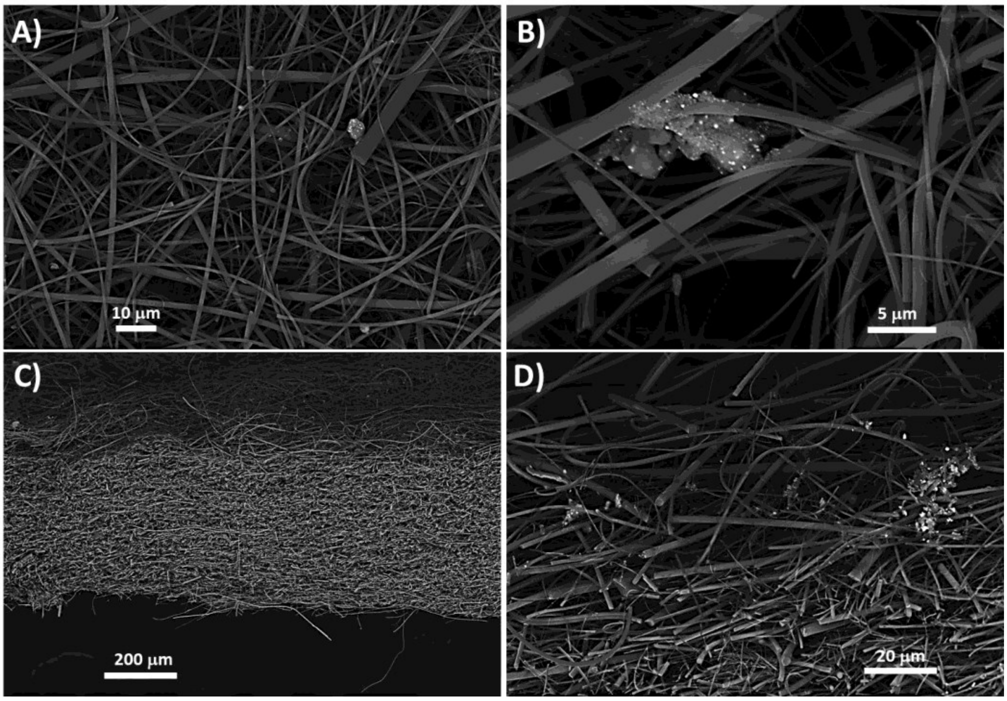

Figure S.4. SEM micrographs (BEI 20kV) of PAS4μg at 1700X (A) and 3500X (B) magnification fibrous substrate with Au@TiO$_2$ NPs clusters; side view (90X) (C) and magnification of the area beneath the surface (430X)(D)

Finally, the most diluted suspension involved an even more sporadic, heterogeneous and disordered distribution of the particles with sparse aggregations of different sizes fastened to the fibers. As expected, no organization between the particles in more complex architectures was observed (Figure S.4.A-D)

S.2. PASs assembling procedure

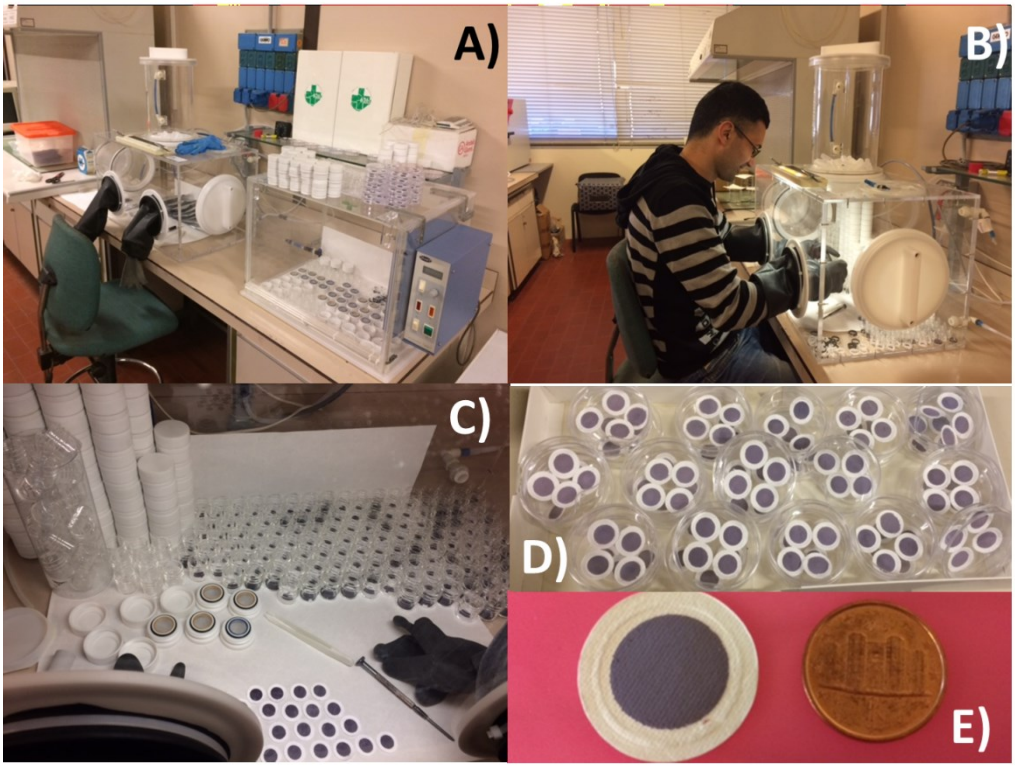

Fig. S.5. A composite picture showing a roundup of some steps in the passive assembly procedure in IIA-CNR Laboratory (Monterotondo, RM, Italy): A) the incubator where the components of the passive samples stop after having been carefully cleaned (*see manuscript for the procedure*), waiting to be mounted into the final devices; B) PASs assembling in a glove box**.** These procedures have to be carried out in a controlled atmosphere (clean air 5.0 flowing through an activated carbon cartridge) to avoid mercury contamination from environment. C) Internal view of the glove-box where passive samples are assembled and sealed; D) small containers with passive membranes coated with TiO2@AuNPs (PAS4mg); E) the adsorbing passive layer size in comparison with 1 euro cent.

The composite Figure S.5 is intended to testify to the development and handling of many dozens of devices, as stated in the manuscript within the laboratory. A more detailed description of the PASs assembling procedure is reported in the paragraph *2.2.2. PAS assemblage* of the original manuscript.

S.3. Monitoring system

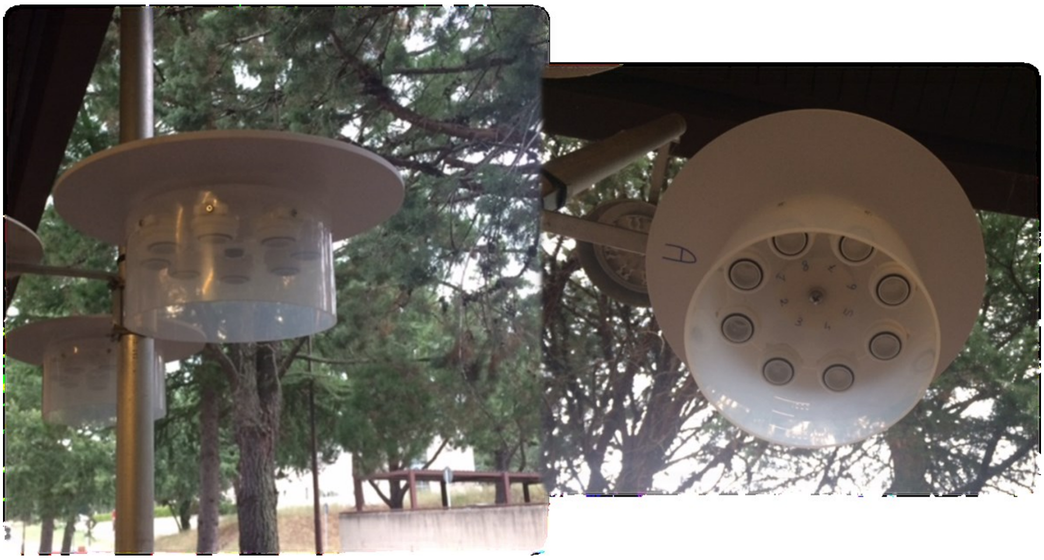

Fig. S.6. A pole sustaining up 3 customized shelters *(on the left) where each could host up to 8 PASs*

The description of the monitoring strategy has been reported in the paragraph *2.2.3. Monitoring system arrangement* of the original manuscript. Figure S.6 shows one of the poles located in the research area of CNR (Monterotondo, Rome, Italy) hosting up to 3 shelters placed at the same distance from the ground. On the right you can observe a bottom view of a shelter housing up to 8 PASs for air monitoring. Five poles were dedicated to the present study.

Authors Affiliations

[1] Laboratory for Biomimetic Membranes and Textiles, Empa, Swiss Federal Laboratories for Materials Science and Technology, Lerchenfeldstrasse 5, CH-9014 St. Gallen, Switzerland; joshua.avossa@empa.ch (JA)
[2] Department of Innovation in Biological Systems, Food and Forestry (DIBAF), Via S. Camillo de Lellis, University of Tuscia, Viterbo, 00100, Italy; decesare@unitus.it (FDC)
[3] Institute of Atmospheric Pollution Research – National Research Council, Research Area of Rome 1, Via Salaria km 23,600, Monterotondo, Rome, 00016, Italy; p.papa@iia.cnr.it (PP); e.zampetti@iia.cnr.it (EZ); a.bearzotti@iia.cnr.it (AB); a.macagnano@iia.cnr.it (AM)
[4] Institute of Chemical Sciences and Technologies "Giulio Natta" (SCITEC) – National Research Council, c/o Area di Ricerca di Milano 1, Via Alfonso Corti 12 - 20133 Milano (MI); marcello.marelli@scitec.cnr.it (MM)
[5] Institute of Atmospheric Pollution Research – National Research Council, Division of Rende, UNICAL Polifuzionale, 87036 Rende (CS), Italy

* Correspondence: antonella.macagnano@cnr.it (AM)
